# Godspeed, *EClinicalMedicine*

**DOI:** 10.1016/j.eclinm.2018.07.004

**Published:** 2018-08-10

Today, we present the first issue of *EClinicalMedicine*, a journal we trust will be a new home for your clinical and public health research. We aspire to identify, attract, and publish original research that supports change and advances knowledge in all areas of medical research, clinical practice, public health, and health policy. Clearly, like other medical journals, we too want to help communicate research that positively impacts the course of human health or helps us to think differently about a specific medical or public health issue. Still, at the heart of our endeavour lies our commitment to research excellence, transparency, and reproducibility. We, of course, appreciate the necessary restrictions on disclosing human data and we place a premium on protecting patient confidentiality. However, in the interest of improving and accelerating research communication and discovery, we strongly encourage our authors to, where possible, make their data publicly available.

We aim to be inclusive. As such, we will also be receptive to quality submissions of timely and important confirmatory or negative data. Irrespective of the conceptual framework of each piece, and as part of *The Lancet* family of journals, our content will be anchored in our commitment to publishing responsible and rigorous work.

*EClinicalMedicine* will maintain a high standard of peer-review and publication, involving continuous and active communication between authors, editors, and reviewers. Based on our collective editorial experience, this process will be a valuable and constructive one where the final product is a much-improved article. But we acknowledge its limitations too. A discussion of the current publishing landscape, interconnected with the strengths and weaknesses of peer-review, will be addressed in a subsequent editorial. Still, it is worthwhile mentioning here that we fully recognise the value of discussions that take place before and after peer-review. As preprints in life sciences and medicine gain more traction and attention, discussions that help shape an article towards publication are no longer limited to conferences or confidential peer-review, but are expanding to include the community and readers more broadly. This diversity of debate offers valuable learning and educational opportunities for everyone. It is also our hope that these global scientific conversations will help to promote women and minorities in science, technology, engineering, and mathematics (STEM) by making their contributions ever more visible. The issue of inequality in STEM is an issue that we, editors from *The Lancet* family of journals, are conscious of and sensitive to. Recent reports have suggested that potential explicit and implicit biases in the work of scientific journals contribute to the under-representation of women and minorities in STEM [1], [2], and we take these findings and their implications very seriously. At *EClinicalMedicine* we are committed to help reverse inequities and inequalities in science and medicine by taking an active role to ensure that both peer-review participation as well as our commissioned content is as gender and geographically balanced as possible.

Long gone are the days when manuscripts arrived by mail at the editor’s office and were then posted to reviewers, who would once again then submit their reports by mail. Today, there are more connected devices than people; yet, due to a multitude of factors, the timeframe from acceptance of a scientific article to its publication is often suboptimal. We appreciate that the landscape of clinical research is fast-moving and that competition for funding is fierce. We acknowledge the importance of making clinical insights available as quickly as possible. By virtue of our digital platform, we have fewer restrictions on the number of papers we can publish. Accepted and proof-corrected articles will be made available online within 48 hours.

An article’s journey does not end with its publication. In fact, it may well be just the start. No article is an island. Every article builds on the knowledge and advances of others, and serves as foundation on which further advances can be constructed. Our task, as professional editors, continues after a peer-reviewed article sees the light of the day. We will engage actively with the research community and our readers to promote our content on diverse social media platforms and to commission Commentaries that capture and contextualise research findings. In doing so, we hope to ensure rapid and wide dissemination of new research findings far beyond those doing science. Ideally, all scientific knowledge should be available to everyone immediately, and everyone should have the same means to communicate their quality research. While science publishing remains a long way from that reality, being an exclusively open access journal means that the impact of the work we publish will not be adversely affected by limiting its readership. Although accepted papers are subject to an article processing change, authors from low-income and middle-income countries with insufficient funding may be eligible for a fee reduction or waiver.

No article is an island and no journal should be one either. We are fortunate to work with the full support and collaboration of the entire *Lancet* team. Through close interaction with *The Lancet* and *The Lancet* specialty titles, we are committed to serve our authors and the scientific community by helping you to find a good home for your clinical research. Through partnership with our sibling journal *EBioMedicine*, whose focus is translational research, we also aim to bridge the communication gap that still exists between laboratory researchers and clinicians. Because technology and medicine are intrinsically connected, improving communication between translational and clinical research is key for fostering collaboration and promoting original thinking that will ultimately benefit society.

As John Glenn was sitting inside the Mercury Friendship 7 capsule in 1962, ready for launch on his journey to circle the globe three times, Scott Carpenter, the back-up astronaut for the mission, wished him a good journey: “Godspeed, John Glenn”—a phrase that almost 60 years later still resonates with the idea of discovery. Although editors don’t get to orbit the world at unimaginable speed, we are thrilled about our journey ahead. Every day, we are privileged to learn something new from our authors and readers, and we promise to work with you to help communicate your important work. It is part of our mission that you too will always discover something new and exciting within our (virtual) pages. Godspeed, *EClinicalMedicine*.
